# Efficacy and safety of pulsed field ablation compared to cryoballoon ablation in the treatment of atrial fibrillation: a meta-analysis

**DOI:** 10.1093/ehjopen/oeae044

**Published:** 2024-05-29

**Authors:** Isabel Rudolph, Giulio Mastella, Isabell Bernlochner, Alexander Steger, Gesa von Olshausen, Franziska Hahn, Reza Wakili, Karl-Ludwig Laugwitz, Eimo Martens, Manuel Rattka

**Affiliations:** School of Medicine and Health, Department of Clinical Medicine—Clinical Department for Cardiology, University Medical Centre, Technical University of Munich, Ismaninger Straße 22, 81675 Munich, Germany; School of Medicine and Health, Department of Clinical Medicine—Clinical Department for Cardiology, University Medical Centre, Technical University of Munich, Ismaninger Straße 22, 81675 Munich, Germany; School of Medicine and Health, Department of Clinical Medicine—Clinical Department for Cardiology, University Medical Centre, Technical University of Munich, Ismaninger Straße 22, 81675 Munich, Germany; School of Medicine and Health, Department of Clinical Medicine—Clinical Department for Cardiology, University Medical Centre, Technical University of Munich, Ismaninger Straße 22, 81675 Munich, Germany; School of Medicine and Health, Department of Clinical Medicine—Clinical Department for Cardiology, University Medical Centre, Technical University of Munich, Ismaninger Straße 22, 81675 Munich, Germany; School of Medicine and Health, Department of Clinical Medicine—Clinical Department for Cardiology, University Medical Centre, Technical University of Munich, Ismaninger Straße 22, 81675 Munich, Germany; Department of Medicine and Cardiology, Goethe University, Frankfurt, Germany; German Centre for Cardiovascular Research (DZHK), partner site Rhine-Main, Germany; School of Medicine and Health, Department of Clinical Medicine—Clinical Department for Cardiology, University Medical Centre, Technical University of Munich, Ismaninger Straße 22, 81675 Munich, Germany; German Centre for Cardiovascular Research (DZHK), partner site Munich, Germany; School of Medicine and Health, Department of Clinical Medicine—Clinical Department for Cardiology, University Medical Centre, Technical University of Munich, Ismaninger Straße 22, 81675 Munich, Germany; European Reference Network Guard Heart, European Union; School of Medicine and Health, Department of Clinical Medicine—Clinical Department for Cardiology, University Medical Centre, Technical University of Munich, Ismaninger Straße 22, 81675 Munich, Germany

**Keywords:** Atrial fibrillation, Pulsed field ablation, Cryoballoon ablation, Pulmonary vein isolation

## Abstract

**Aims:**

Pulmonary vein isolation (PVI) represents the gold standard in the treatment of atrial fibrillation (AF) and the use of single-shot techniques, such as cryoballoon ablation (CBA) and pulsed field ablation (PFA) using a pentaspline catheter, has gained prominence. Recent studies hypothesize that PFA might be superior to CBA, although procedural efficacy and safety data are inconsistent. A meta-analysis was conducted to compare both energy sources for the treatment of AF.

**Methods and results:**

A structured systematic database search and meta-analysis were performed on studies investigating outcomes, periprocedural complications, and/or procedural parameters of AF patients treated by either CBA or PFA. Eleven studies reporting data from 3805 patients were included. Pulmonary vein isolation by PFA was associated with a significantly lower recurrence of atrial fibrillation/atrial tachycardia [odds ratio (OR) = 0.73, 95% confidence interval (CI) = 0.54–0.98, I^2^ = 20%] and fewer periprocedural complications (OR = 0.62, 95% CI = 0.40–0.96, I^2^ = 6%) compared to CBA. The lower complication rate following PFA was mainly driven by fewer phrenic nerve injuries (OR = 0.19, 95% CI = 0.08–0.43, I^2^ = 0%). However, there were more cases of cardiac tamponades after PFA (OR = 2.56, 95% CI = 1.01–6.49, I^2^ = 0%). Additionally, using PFA for PVI was associated with shorter total procedure times [mean difference (MD) = −9.68, 95% CI = −14.92 to −4.43 min, I^2^ = 92%] and lower radiation exposure (MD = −148.07, 95% CI = −276.50 to −19.64 µGy·mI^2^ = 7%).

**Conclusion:**

Our results suggest that PFA for PVI, compared to CBA, enables shorter procedure times with lower arrhythmia recurrence and a reduced risk of periprocedural complications. Randomized controlled trials need to confirm our findings.

## Introduction

In the context of managing atrial fibrillation (AF), catheter ablation has emerged as a crucial therapeutic approach aimed at maintaining sinus rhythm and alleviating symptoms in affected individuals.^1^ Based on the observation that arrhythmogenic triggers originating from pulmonary vein muscle sleeves contribute to the initiation of AF, the electrical isolation of pulmonary veins (PVI) through ablation has become a central component of interventional rhythm control therapy.^1^ Among the various techniques utilized, single-shot devices have garnered attention due to their procedural simplicity.^2^ In addition to the established single-shot procedure for PVI using cryoballoon ablation (CBA), catheters using pulsed field ablation (PFA) for single-shot PVI have recently emerged as promising new ablation modalities.^3^ Pulsed field ablation harnesses electrical fields to induce electroporation, allowing for a precise targeting of cardiac tissue, thereby potentially reducing damage to the neighbouring anatomic structures.^4^ Cryoballoon ablation is a well-established technique that employs low temperatures to create lesions via thermal cell damage to the area surrounding the pulmonary veins, thereby interrupting their electrical connection to the atrial myocardium.^2^ While both approaches have shown promising results in treating AF, studies directly comparing their safety profiles and effectiveness are sparse.^5,6^ Since both a safe and effective ablation is desired, the necessity of conducting a comparative analysis of these techniques is increasingly evident. This meta-analysis seeks to systematically compare the safety and efficacy of PFA and CBA in the treatment of AF.

## Methods

### Data sources and study selection

**Figure 1:**
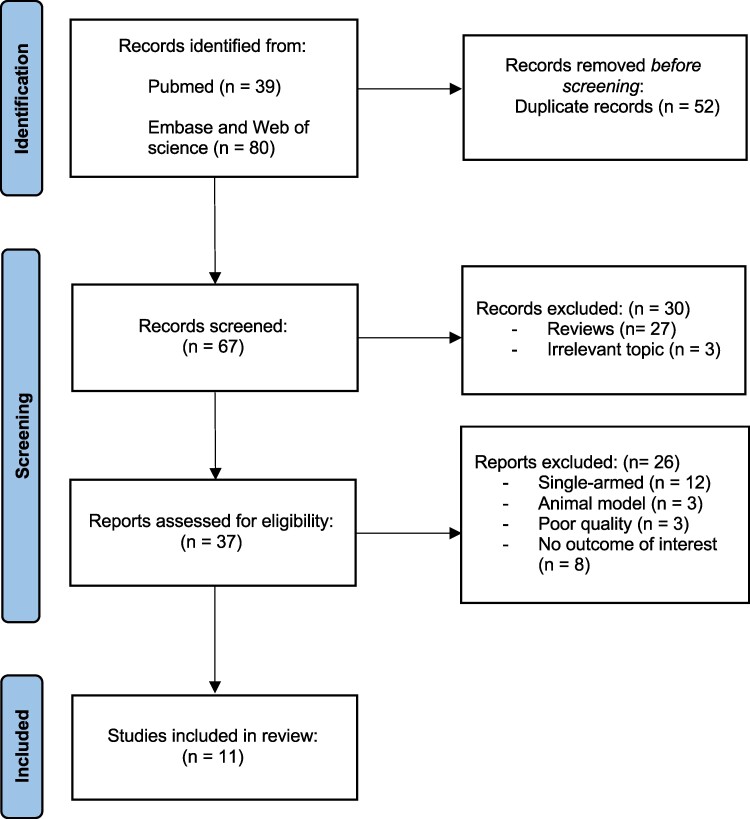
PRISMA flow diagram. Out of 119 identified studies and after application of the inclusion and exclusion criteria, 11 studies were included in the quantitative synthesis.

A comprehensive literature search was performed through PubMed, Embase, and Web of Science through 31 January 2024 for studies comparing outcomes of AF patients treated by either CBA or PFA using the keywords ‘cryoballoon ablation’ and ‘pulsed field ablation’. We searched for randomized controlled studies, observational studies, and register-based studies, without language restrictions. Conference abstracts, case reports, review articles, and posters were excluded (see Supplementary material online, *Table S1*). The flowchart illustrating the literature search strategy is shown in *Figure 1*.

### Data extraction and study quality

Two investigators independently reviewed all the articles, selected eligible studies and extracted valuable data. In case of any discrepancies, a third reviewer made the final decision. Data extraction used standardized extraction forms including information on authors, country of origin, publication year, number of patients per group, number of patients with freedom from atrial fibrillation/atrial tachycardia (AF/AT) at the end of follow-up, number of complications [any access site complications, transient and/or persistent phrenic nerve injury, cardiac tamponade, any oesophageal injury, transient ischaemic attack (TIA) and/or stroke], total procedure time, total fluoroscopy time, total radiation dose, amount of contrast dye used during the procedure, and baseline characteristics (age, gender, type of atrial fibrillation, number of patients with a history of TIA/stroke, arterial hypertension, diabetes mellitus, known coronary artery disease). The study quality was assessed as detailed by the NIH Quality Assessment Tool and studies were rated as being of either ‘good’, ‘fair’, or ‘poor’ quality.^7^ Studies rated as being of ‘poor’ quality were excluded from further analysis. As all analyses were based on previous studies, neither patient consent nor ethical committee approval was required for our study. This meta-analysis has been pre-registered at PROSPERO (CRD 42024502083).

### Outcomes

We sought to compare efficacy and safety of PFA vs. CBA for AF treatment. Our studies efficacy outcome was AF/AT freedom at the end of follow-up. The safety outcome included the frequencies of any access site complications, transient and/or persistent phrenic nerve injury, cardiac tamponade, any oesophageal injury, TIA, and/or stroke. Additionally, total procedure time, total fluoroscopy time, and total radiation dose were compared.

### Statistical analysis

Data were collected in a Microsoft Excel® spreadsheet and outcome measures were calculated. If in the included studies only median values with interquartile ranges were reported, means and standard deviations were calculated by making use of the Box–Cox method.^8^ Meta-analyses were done both on binary and on continuous endpoints. The data are reported as (1) absolute frequencies and/or percentages in case of binary endpoints and (2) as mean ± standard deviations (SD) in case of continuous endpoints. The random effects model was used to combine the estimates from the different studies. This was preferred over the fixed effects model since the random effect model gives a more conservative estimate. For binary endpoints, the Mantel–Haenszel method was used, and the odds ratio (OR) incl. 95% confidence interval is reported as effect measure. For continuous endpoints, the inverse variance method was used, and the mean difference (MD) incl. 95% confidence interval is reported as an effect measure. For the assessment of heterogeneity among studies, the statistical characteristic I^2^ is reported. Forest plots are used for graphical representation of results, which included all individual results of the considered studies separately, as well as overall results. Additionally, funnel plots were created to evaluate the risk of publication bias. Meta-analyses were performed using the meta package in the statistical software R, version 3.5.1. All tests were two-tailed, and results with a *P* value of <0.05 were considered statistically significant. The Preferred Reporting Items for Systematic Reviews and Meta-Analyses (PRISMA) statement was applied in this study.^9^

## Results

### Literature search, quality assessment, and baseline characteristics

Based on our search strategy, 119 studies were searched from PubMed, Embase, and Web of Science databases. After removal of 53 duplicates and exclusion of 30 studies that were either reviews or not related to the research question, 37 articles were assessed for eligibility. Finally, 11 studies were included in the quantitative synthesis (*Figure 1*).^5,6,10–18^ Based on the NIH Study Quality Assessment tool, nine studies had ‘good’, and two studies ‘fair’ methodical quality (*Table 1*). A total of 3805 patients were enrolled from the various studies, including 1531 patients undergoing PVI by PFA and 2274 patients treated by CBA. In order to conduct PFA, the procedural characteristics for all studies encompassed eight applications per pulmonary vein, with minor variations for CBA (see Supplementary material online, *Table S2*). Evaluation of baseline characteristics showed that the average age was 65 years with 66% of patients being male. 67% of patients were treated for paroxysmal AF. Detailed information on baseline characteristics is listed in *Table 1*.

**Table 1:** Summary of included studies and baseline characteristics

Study	Country	NIH	No. of patients	Age (mean, years)	Sex, males (%)	HTN (%)	DM (%)	S/T (%)	CAD (%)	PAF (%)
Badertscher *et al*.^14^	Switzerland	Good	181	64	64	55	10	n/a	8	64
Della Rocca al.^15^	Multinational	Good	522	62	63	44	8	5	7	100
Grosse Meininghaus *et al*.^12^	Germany	Fair	53	68	58	91	15	n/a	n/a	43
Maurhofer *et al*.^11^	Switzerland	Good	120	63	73	63	9	5	18	100
Rattka *et al*.^16^	Germany	Good	141	63	66	70	22	6	n/a	55
Reddy *et al*.^5^	Multinational	Good	440	62.4	65	54	11	4	12	100
Schipper *et al*.^10^	Germany	Good	108	68	69	70	17	n/a	29	31
Kupusovic *et al*.^17^	Germany	Fair	26	65	81	85	12	8	n/a	50
Urbanek *et al*.^6^	Germany	Good	400	68	57	68	15	6	14	61
van de Kar *et al*.^18^	Netherlands	Good	1714	63	68	n/a	8	n/a	n/a	65
Wahedi *et al*.^13^	Germany	Good	100	66	61	65	11	5	20	65

NIH, study quality as per NIH Quality Assessment Tool; HTN, arterial hypertension; DM, diabetes mellitus; S/T, stroke/transitory ischaemic attack; CAD, coronary artery disease; PAF, paroxysmal atrial fibrillation; n/a, data not available.

### Efficacy and safety outcome

**Figure 2:**
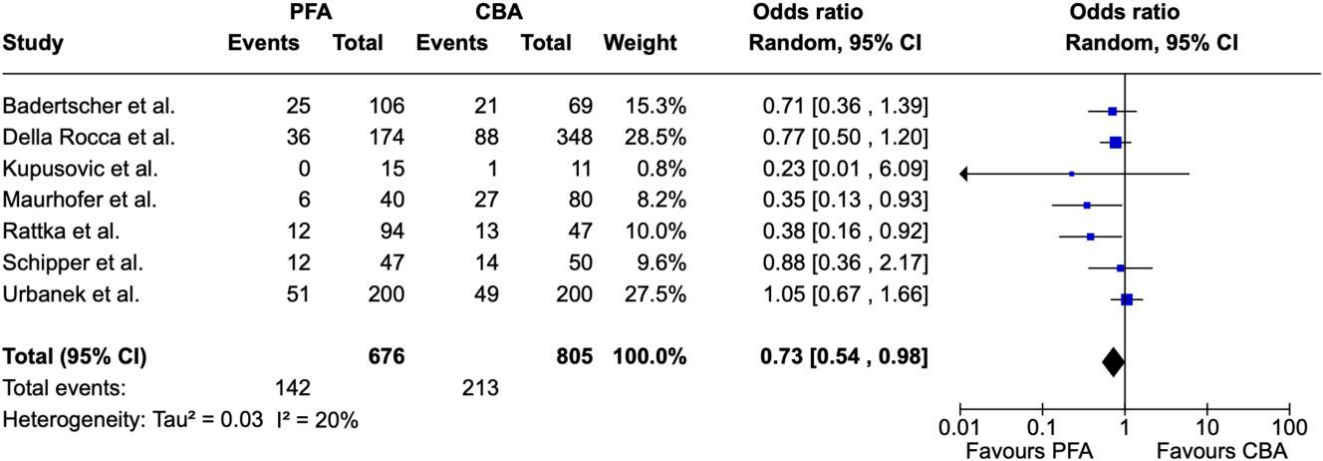
Forest plot—arrhythmia recurrence. Atrial fibrillation/atrial tachycardia recurrence was significantly higher after pulsed field ablation. PFA, pulsed field ablation; CBA, cryoballoon ablation; CI, confidence interval.

Procedural efficacy was assessed by the number of AF/AT recurrence at the end of follow-up and could be extracted from seven studies, all of which included patients undergoing PVI for the first time. Remarkably, arrhythmia recurrence was significantly lower in patients undergoing PVI by PFA compared to CBA (OR = 0.73, 95% CI = 0.54–0.98, I^2^ = 20%; *Figure 2*).

**Figure 3:**
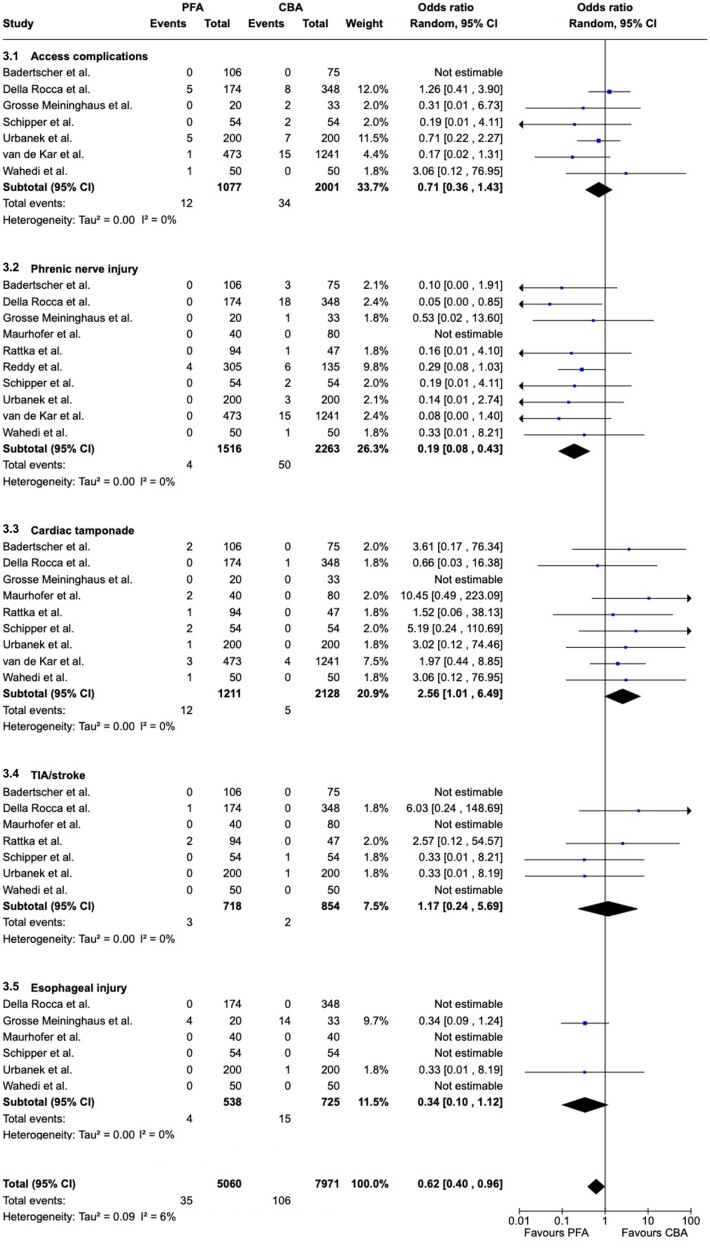
Forest plot—periprocedural complications. Overall, complications were significantly lower in the pulsed field ablation (PFA) group. Phrenic nerve injuries were significantly lower in patients undergoing PFA (3.2), while cardiac tamponades were more frequent if PFA for pulmonary vein isolation was performed (3.3). Otherwise, there were no significant differences in the subgroup analyses. PFA, pulsed field ablation; CBA, cryoballoon ablation; CI, confidence interval; TIA, transitory ischaemic attack.

With regard to safety, periprocedural complications, which were reported in 10 studies, were significantly lower in the PFA group (OR = 0.62, 95% CI = 0.40–0.96, I^2^ = 6%; *Figure 3*). As for the subgroups, there was a significant difference for phrenic nerve injuries. Patients treated by PFA suffered significantly fewer phrenic nerve injuries compared to those undergoing CBA (OR = 0.19, 95% CI = 0.08–0.43, I^2^ = 0%; *Figure 3.2*). Additionally, there was a trend towards fewer oesophageal injuries in the PFA group (OR = 0.34, 95% CI = 0.10–1.12, I^2^ = 0%; *Figure 3.5*). Remarkably, we observed significantly more cardiac tamponades after PFA (OR = 2.56, 95% CI = 1.01–6.49, I^2^ = 0%; *Figure 3.3*). For vascular access complications (OR = 0.71, 95% CI = 0.36–1.43, I^2^ = 0%) and TIA/stroke (OR = 1.17, 95% CI = 0.24–5.69, I^2^ = 0%; *Figure 3.1* and *3.4*), there were no significant differences. Corresponding, funnel plots are shown as supplementary material online, *Figures S1* and *S2*.

### Procedure time, fluoroscopy time, and radiation dose

**Figure 4:**
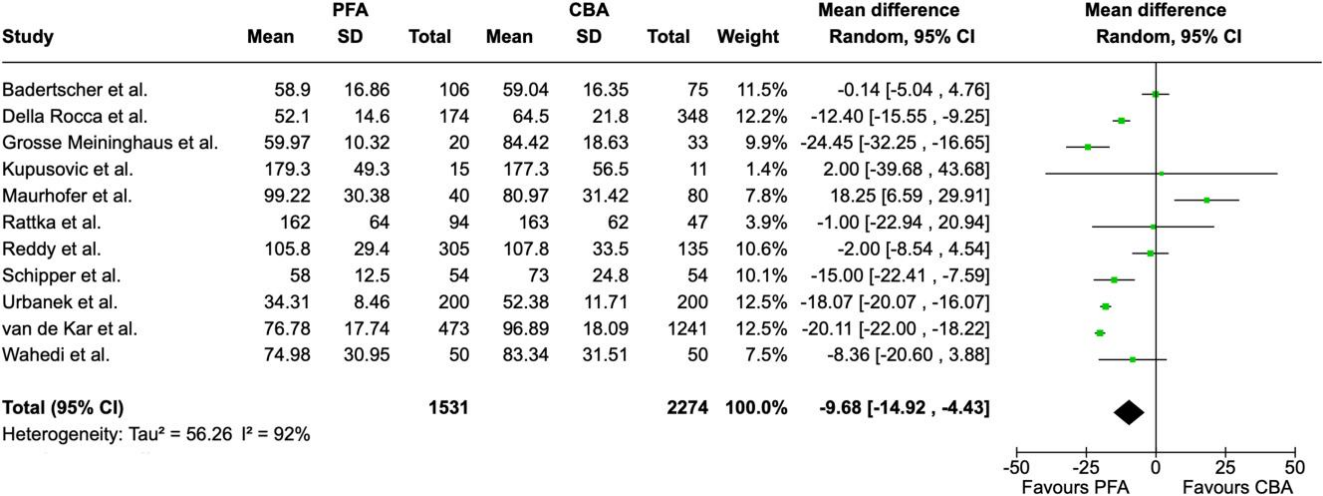
Forest plot—total procedure time. Total procedure time was significantly lower in the pulsed field ablation group. PFA, pulsed field ablation; CBA, cryoballoon ablation; CI, confidence interval.

**Figure 5:**
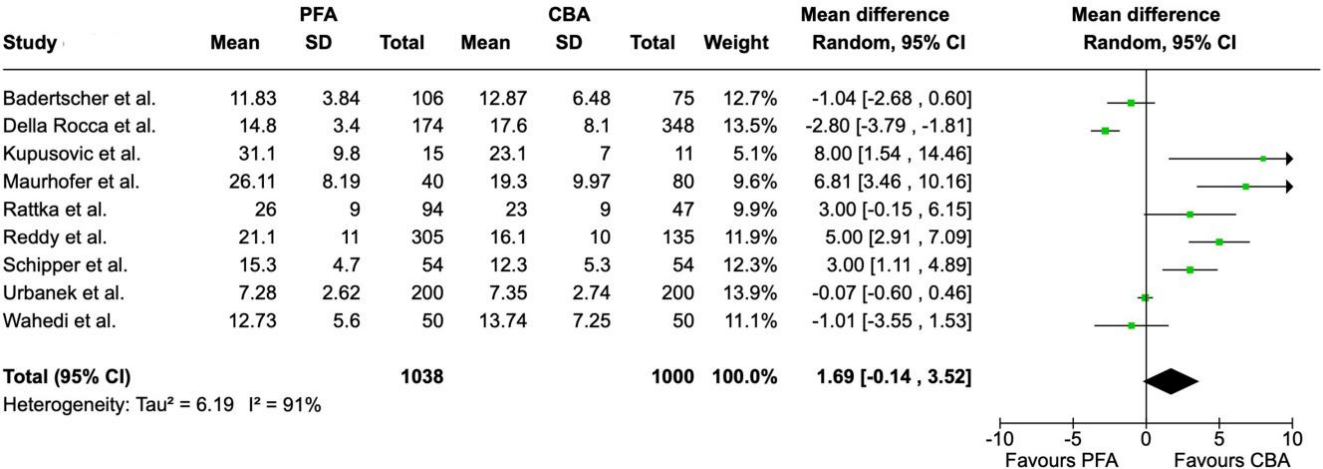
Forest plot—fluoroscopy time. For fluoroscopy time, there was no significant difference between both groups. PFA, pulsed field ablation; CBA, cryoballoon ablation; CI, confidence interval.

**Figure 6:**
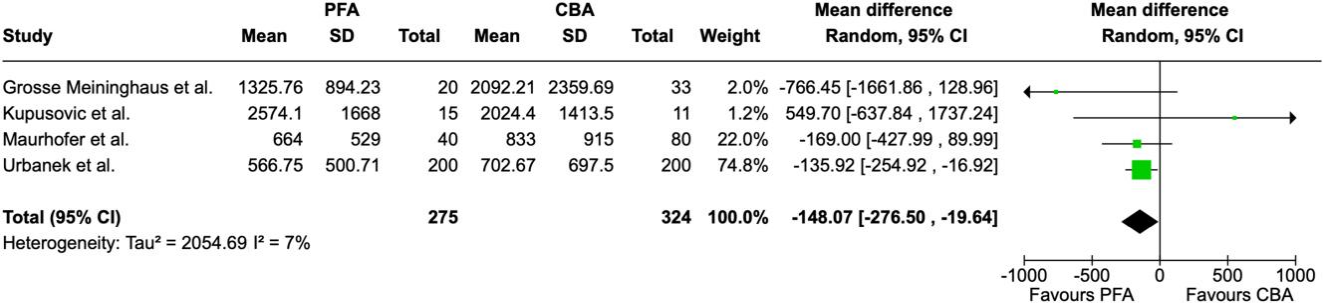
Forest plot—radiation dose. Radiation dose was significantly lower in patients treated by pulsed field ablation. PFA, pulsed field ablation; CBA, cryoballoon ablation; CI, confidence interval.

Total procedure time was reported in 11 studies. We found that PFA procedures had a significantly shorter duration compared to CBA procedures (MD = −9.68, 95% CI = −14.92 to −4.43 min, I^2^ = 92%; *Figure 4*). Fluoroscopy time, which was reported in nine studies, did not differ significantly between groups (MD = 1.69, 95% CI = −0.14 to −3.52 min, I^2^ = 91; *Figure 5*). Radiation dose was extracted from 4 studies and was found to be significantly lower in patients undergoing PFA (MD = −148.07, 95% CI = −276.50 to −19.64 µGy·m^2^ , I^2^ = 7%; *Figure 6*). Supplementary material online, *Figures S2*–*S5* display funnel plots.

## Discussion

In this comprehensive meta-analysis encompassing 11 trials and involving a total of 3805 patients, a comparative assessment was conducted on the efficacy and safety of PFA and CBA for PVI in patients diagnosed with AF. The key findings of this study are as follows: (1) Patients undergoing PFA had significantly lower rates of AF/AT recurrence; (2) overall complications were significantly lower post-PFA, (3) primarily attributed to a decrease in phrenic nerve injuries. Interestingly, (4) there were more cardiac tamponades in the PFA group. Additionally, (5) the total procedure time was notably shorter for individuals treated by PFA.

Pulsed field ablation has emerged as an innovative technique utilizing alternating high-intensity electric fields to induce irreversible electroporation within cardiac tissues.^3^ This non-thermal methodology sets PFA apart from traditional ablation techniques like CBA. Cryoballoon ablation, developed as an alternative to point-by-point radiofrequency ablation (RFA), uses nitrous oxide gas as a cooling agent to create circumferential lesions around the pulmonary veins.^2^ Since comparative data on the efficacy and safety of both ablation modalities are sparse, we conducted a meta-analysis to improve insights on this important topic.

In patients afflicted with paroxysmal AF, thermal ablation modalities, such as CBA and RFA, typically achieve freedom from AF/AT in 70–80% of cases within a one-year timeframe.^2^ The ADVENT-trial, which compared PFA and conventional thermal ablation in patients with paroxysmal AF, unveiled a one-year success rate of 73% for PFA recipients and 71% for those subjected to thermal ablation, demonstrating that PFA is not inferior when compared to the established standard treatment.^5^ Nevertheless, to date, there are no randomized controlled trials directly comparing the efficacy of PFA and CBA in a 1:1 fashion. In our meta-analysis, we observed a significantly reduced recurrence of AF/AT in patients undergoing PFA. Of the seven studies detailing AF/AT frequencies, six indicated a lower recurrence following PVI by PFA.^10,11,14–17^ The underlying cause remains subject to debate. A pre-clinical study assessing the durability of lesions induced by the pentaspline PFA catheter disclosed a PVI durability rate of 96%.^4^ In contrast, CBA exhibits a lower durability rate at 81%, hinting towards the potential superiority of PFA in generating more substantial and lasting PVIs than CBA.^19^ Consequently, our finding of decreased AF/AT recurrence underscores the proposition that employing PFA for PVI may confer benefits over CBA in managing AF and supports the current trend to switch from the conventional thermal ablation methods towards the new, non-thermal ablation modality. Nonetheless, randomized controlled trials designed with adequate statistical power are indispensable to substantiate this hypothesis.

One of the key advantages of the PFA technology lies in its ability to selectively target specific tissues.^4,20^ This feature helps to minimize damage to adjacent structures, thereby reducing the risk of complications such as phrenic nerve palsy and potentially life-threatening oesophageal fistulas. Most studies included in our meta-analysis did not find significant differences in complication rates between PFA and CBA procedures. Only van de Kar *et al*. noted a lower rate of phrenic nerve palsy with PFA, while Maurhofer *et al*. reported a higher occurrence of cardiac tamponades following PFA treatment.^11,18^ Our meta-analysis indicated a significantly lower rate of complications, particularly phrenic nerve injuries, in patients undergoing PVI by PFA compared to CBA. Furthermore, there was a tendency towards fewer oesophageal lesions with the use of non-thermal energy, although this trend was not statistically significant. For TIA/stroke and vascular access complications we did not observe any significant differences. Consequently, despite previous reports suggesting limited thermal effects associated with PFA, its relative tissue selectivity seems to confer a more favourable safety profile compared to CBA.^20^ Nonetheless, our analysis identified a higher rate of cardiac tamponades following PFA, which could be attributed to various factors. For instance, some studies reported data from patients treated during the introductory phase of the FARAPULSE™ system, implying a learning curve effect. Additionally, many centres initially used a stiff guidewire with a straight tip for navigating the pulmonary veins, later transitioning to a J-tip for safety considerations.^14^ Additionally, the FARADRIVE™ steerable sheath might pose increased risk of trauma due to its outer diameter of 16.8F. While there is no clear evidence suggesting that cardiac tamponades are specific to PVI by PFA, forthcoming research should prioritize this critical safety aspect.

Our analysis revealed significantly shorter procedure durations with PFA compared to CBA. Apart from potential reductions in expenses and enhanced staff satisfaction, shorter procedure times have clinical implications, such as the reduction of postoperative cognitive dysfunction.^21^ The utilization of PFA for PVI represents an innovative technique. Due to its novelty, extended procedure durations are to be anticipated. This might be attributed to the learning curve associated with PFA and the utilization of pre- and post-ablation 3D-mapping techniques to ensure successful PVI upon completion of the PFA procedure. Certain studies within our meta-analysis compared procedure times for PFA with mapping against those for CBA without mapping, thereby influencing our analysis.^11^ Despite these potential biases, our analysis revealed that the overall duration of procedures was notably shorter in the PFA cohort. This difference might be explained by the intricate positioning sometimes required for optimal pulmonary vein occlusion prior to freezing during CBA, as well as the relatively long freezing time.^6^ We did not observe a difference in fluoroscopy time between the two techniques, indicating that both rely on fluoroscopic guidance for navigation within the left atrium. However, it is worth mentioning that some electrophysiologists may adjust the frame rate during contrast injection when evaluating pulmonary vein occlusion, a step that is rendered unnecessary during PFA-based PVIs. Nevertheless, this remains speculative.

## Limitations

As this is a meta-analysis of studies mostly containing observational retrospective studies, it inherently has limitations. As per our pre-specified study protocol data on the amount of contrast dye used during the procedure and number of first pass isolations were extracted but were not sufficient to be included in the analysis. Some studies reported skewed data on total procedure time, fluoroscopy time, and radiation dose, which has been adjusted appropriately.^8^ Heterogeneity as measured by I^2^ was relatively high for total procedural time and fluoroscopy time. This is supposedly related to the difference in workflows and definitions. For instance, Van de Kar *et al*. defined procedure time as the time between arrival and departure from procedure room, while Della Rocca *et al*. defined it as the time measured from femoral puncture to catheter removal. Thus, both results must be interpreted with care. Additionally, differences in ablation protocols in between studies, such as cryoablation strategies, type of anaesthesia, and period of follow-up contribute to heterogeneity (see Supplementary material online, *Table S2*). Nevertheless, for our main outcomes (arrhythmia recurrence and total complications), heterogeneity was low suggesting low variability between studies. Furthermore, arrhythmia recurrence was estimated by the total number of AF/AT recurrence at the end of follow-up and did not consider the time to recurrence. Additionally, in the studies included in our analysis, only the FARAPULSE™ PFA system was used. Consequently, our results are not transferable to other PFA systems. However, all available data comparing rhythm and safety outcomes of AF patients treated by either PFA or CBA for PVI through 31 January 2024, as identified by our search protocol were included. Thus, we believe that this meta-analysis provides an unbiased insight on the comparison of both ablation modalities.

## Conclusion

Our findings indicate that pulmonary vein isolation (PVI) using pulsed field ablation (PFA) may exhibit greater efficacy in preventing arrhythmia recurrence compared to cryoballoon ablation (CBA) for PVI. Furthermore, the relatively higher tissue specificity of PFA appears to contribute to a more favourable safety profile. Nevertheless, although probably not directly attributable to the ablation technique itself, our observation of an elevated incidence of cardiac tamponades in the PFA cohort warrants attention in future investigations. Randomized controlled trials are necessary to validate our results.

## Supplementary Material

oeae044_Supplementary_Data

## Data Availability

All relevant data are within the manuscript and supporting information files.
