# Supplementary material for: Efficacy and safety of pulsed field ablation compared to cryoballoon ablation in the treatment of atrial fibrillation: a meta-analysis
Source: Eur Heart J Open. 2024 May 29;4(3):oeae044. doi: 10.1093/ehjopen/oeae044 (PMC11200106; doi:10.1093/ehjopen/oeae044)
Supplement: oeae044_Supplementary_Data [file oeae044_supplementary_data.zip › results search strategy - supplementary table 1.docx]

| Supplementary table 1. Additional study characteristics | | | | | |
| --- | --- | --- | --- | --- | --- |
| Study | Study type | Follow-up (months) | Blanking time (months) | Type of anaesthesia | CB application |
| Badertscher et al.^14^ | prospective registry | 12 | 3 | CS | 1x240 or 180s if TTI<60s |
| Della Rocca al.^15^ | retrospective | 12 | 3 | GA | 1x240s or 180s if TTI<60s |
| Grosse Meininghaus et al.^12^ | retrospective | N/A | N/A | CS | N/A |
| Maurhofer et al.^11^ | prospective registry | 12 | 3 | CS | TTI + 120s |
| Rattka et al.^16^ | retrospective | 12 | 3 | CS | 1x180s, no TTI |
| Reddy et al.^5^ | RCT | 12 | 3 | GA or CS | OD |
| Schipper et al.^10^ | retrospective | 12 | 3 | CS | 1x240s or 180s if TTI<60s |
| Kupusovic et al.^17^ | retrospective | 6 | 3 | CS | 2x240s, no TTI |
| Urbanek et al.^6^ | retrospective | 12 | 3 | CS | 2x240s or  1x240s if TTI <75s |
| van de Kar et al.^18^ | retrospective | N/A | N/A | N/A | 1x240s |
| Wahedi et al.^13^ | prospective | N/A | N/A | CS | 180-240s  operator dependent, TTI was registered |
| CB, cryoballoon; CS, conscious sedation; GA, general anesthesia; TTI, time to isolation; s, seconds; RCT, randomized controlled trial; OD, operator discretion; n/a, data not available | | | | | |
