# Supplementary material for: Efficacy and safety of pulsed field ablation compared to cryoballoon ablation in the treatment of atrial fibrillation: a meta-analysis
Source: Eur Heart J Open. 2024 May 29;4(3):oeae044. doi: 10.1093/ehjopen/oeae044 (PMC11200106; doi:10.1093/ehjopen/oeae044)
Supplement: oeae044_Supplementary_Data [file oeae044_supplementary_data.zip › Supplementary Figures.pdf]

Supplementary Figure 1

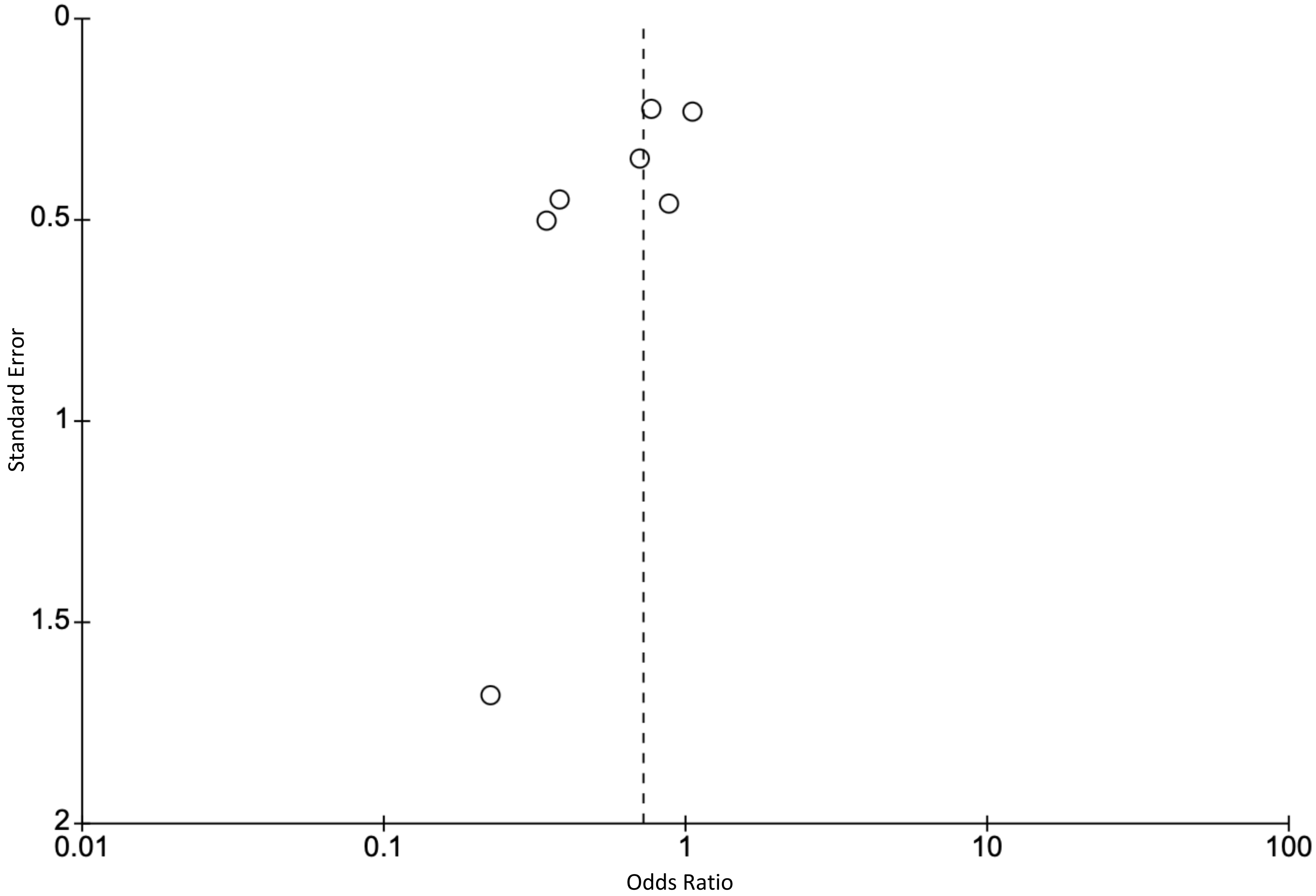

Funnel plot – Arrhythmia recurrence

Supplementary Figure 2

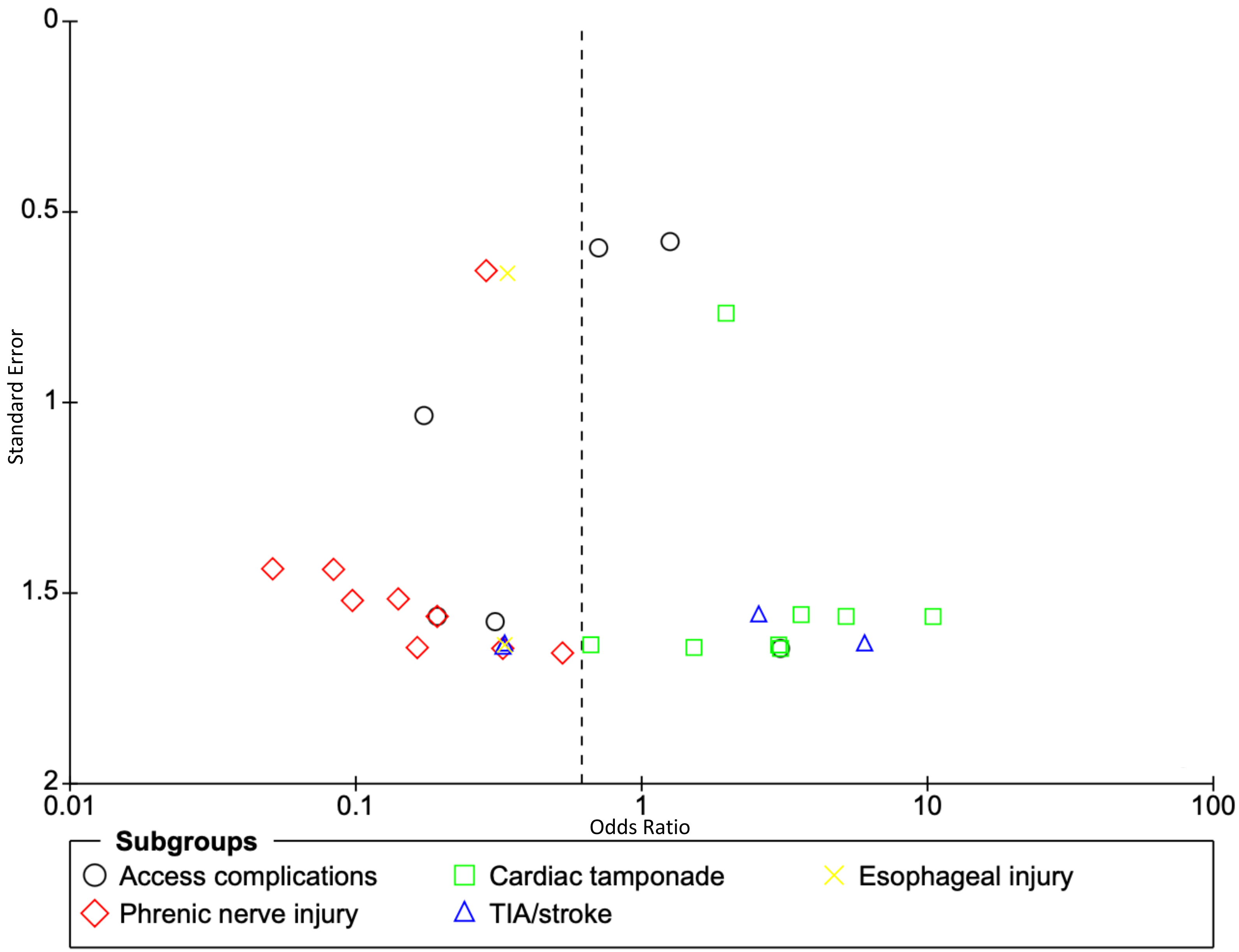

Funnel plot – Periprocedural complications

Supplementary Figure 3

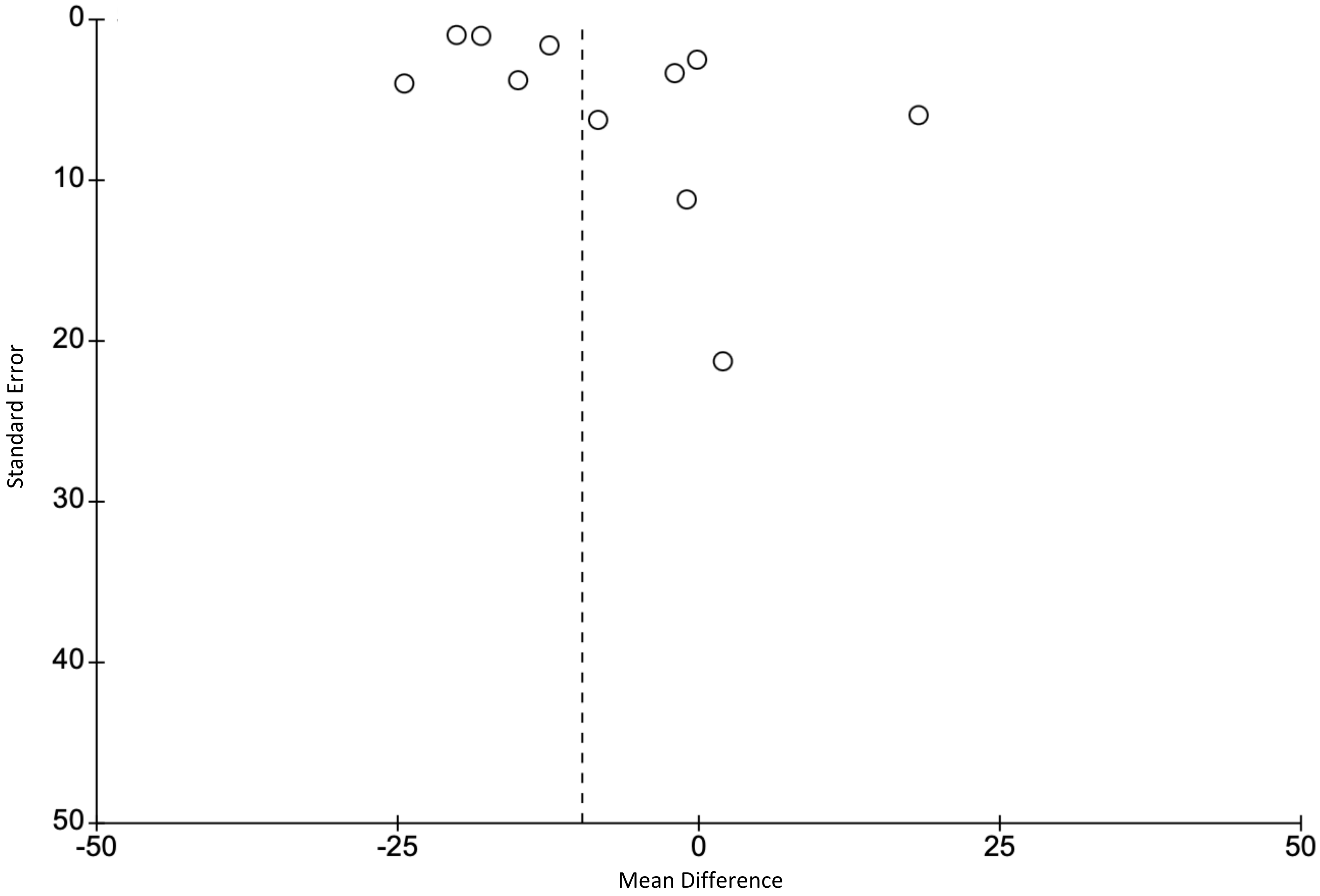

Funnel plot – Total procedure time

Supplementary Figure 4

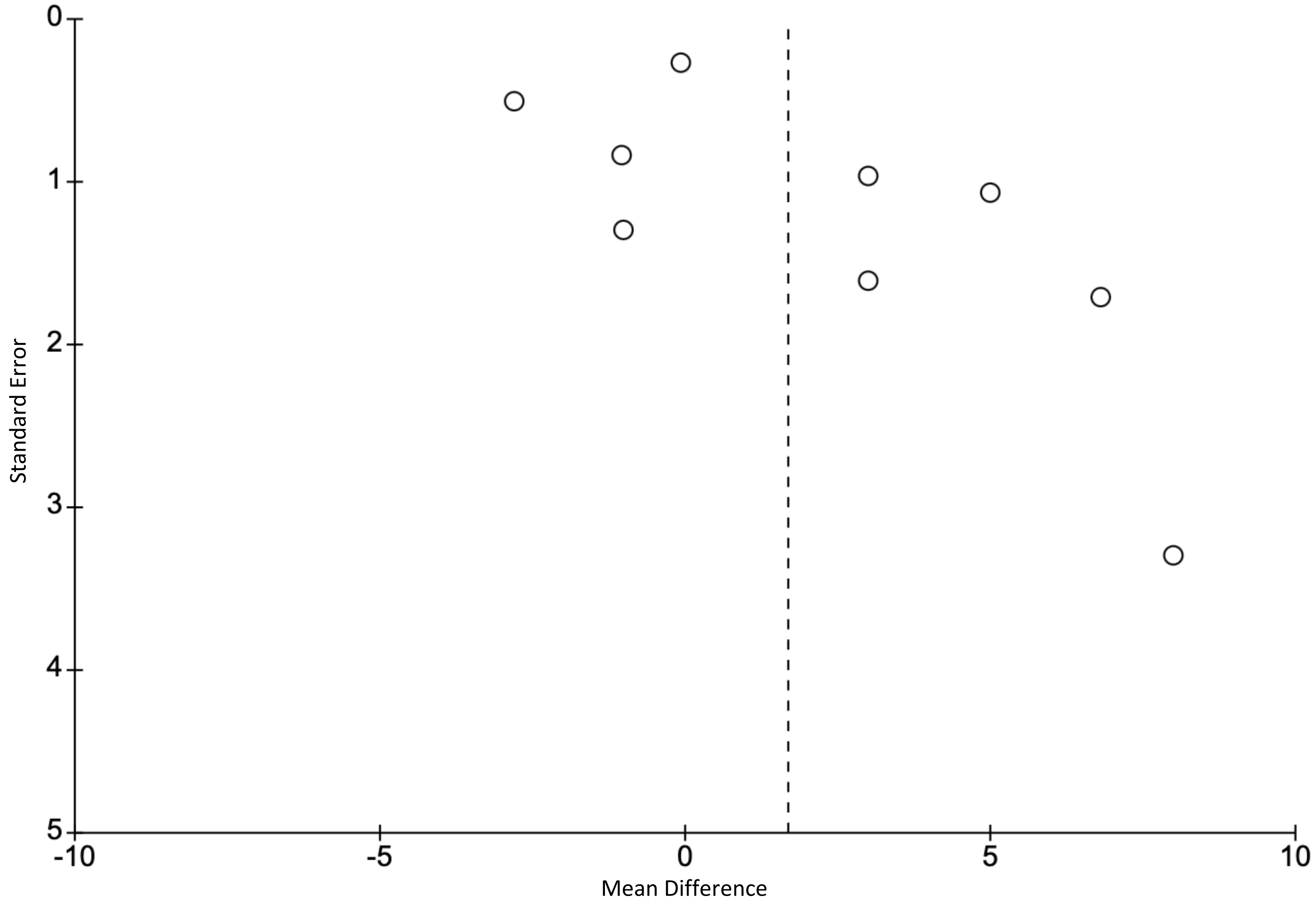

Funnel plot – Fluoroscopy time

Supplementary Figure 5

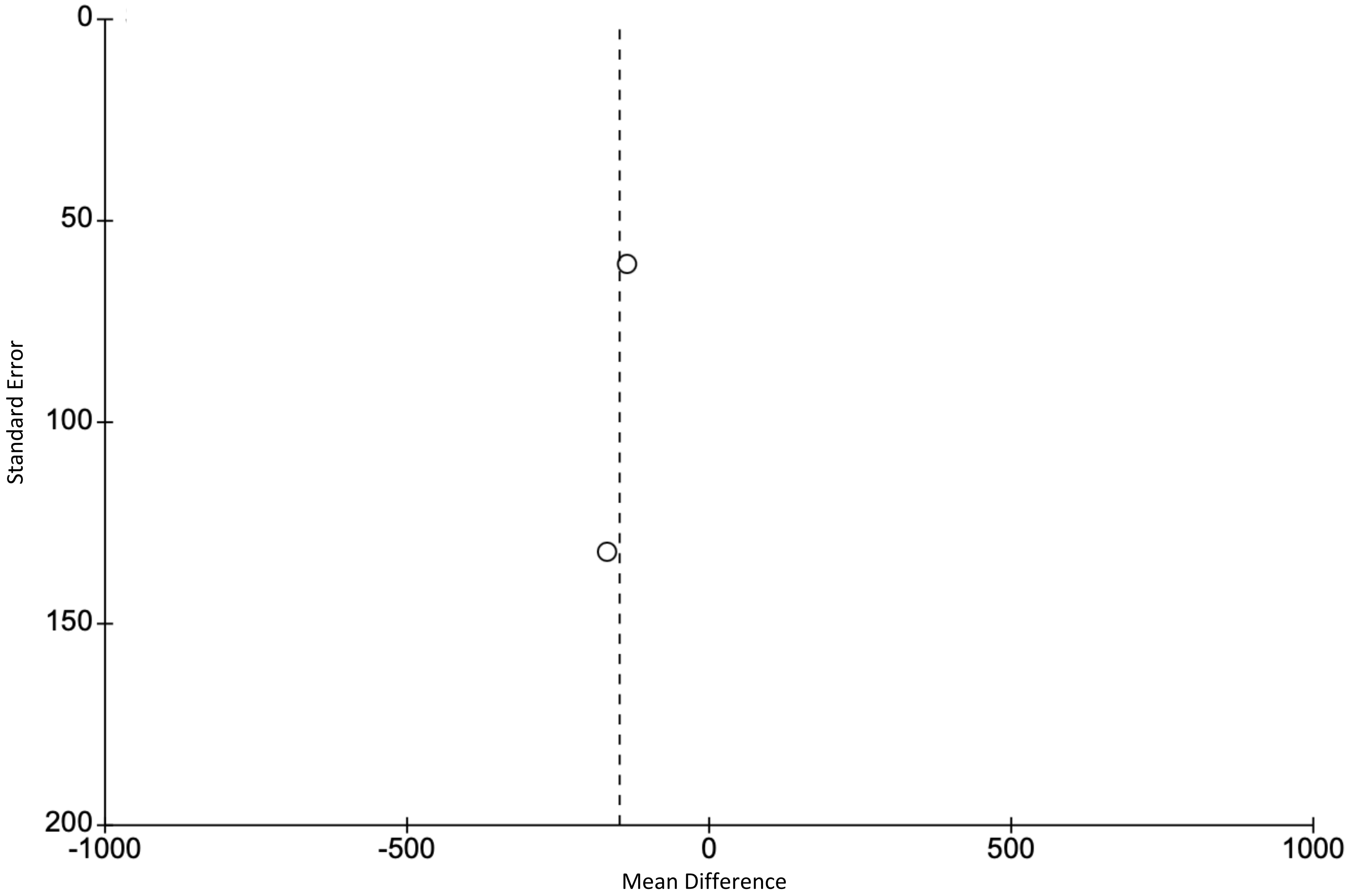

Funnel plot – Radiation dose
